# Integrity of the *Saccharomyces cerevisiae* Rpn11 Protein Is Critical for Formation of Proteasome Storage Granules (PSG) and Survival in Stationary Phase

**DOI:** 10.1371/journal.pone.0070357

**Published:** 2013-08-06

**Authors:** Rémy Saunier, Michela Esposito, Emmanuel P. Dassa, Agnès Delahodde

**Affiliations:** Univ Paris-Sud, CNRS UMR 8621, Institut de Génétique et Microbiologie, Orsay, France; Texas A&M University, United States of America

## Abstract

Decline of proteasome activity has been reported in mammals, flies and yeasts during aging. In the yeast *Saccharomyces cerevisiae*, the reduction of proteolysis in stationary phase is correlated with disassembly of the 26S proteasomes into their 20S and 19S subcomplexes. However a recent report showed that upon entry into the stationary phase, proteasome subunits massively re-localize from the nucleus into mobile cytoplasmic structures called proteasome storage granules (PSGs). Whether proteasome subunits in PSG are assembled into active complexes remains an open question that we addressed in the present study. We showed that a particular mutant of the *RPN11* gene (*rpn11-m1*), encoding a proteasome lid subunit already known to exhibit proteasome assembly/stability defect *in vitro*, is unable to form PSGs and displays a reduced viability in stationary phase. Full restoration of long-term survival and PSG formation in *rpn11-m1* cells can be achieved by the expression *in trans* of the last 45 amino acids of the C-terminal domain of Rpn11, which was moreover found to co-localize with PSGs. In addition, another *rpn11* mutant leading to seven amino acids change in the Rpn11 C-terminal domain, which exhibits assembled-26S proteasomes, is able to form PSGs but with a delay compared to the wild type situation. Altogether, our findings indicate that PSGs are formed of fully assembled 26S proteasomes and suggest a critical role for the Rpn11 protein in this process.

## Introduction

The vast majority of cells from prokaryotes to multicellular organisms exist in a non-dividing state called quiescence, a cellular state defined as a temporary and reversible absence of proliferation. In yeast, the quiescent state can be induced by nutrient starvation as in stationary phase and quiescent cells can support long-term survival [1]. Exit from the quiescent state occurs when a carbon source become available. It has been described that quiescence in yeast cells induces morphological and physiological changes such as a decreased metabolic rate, an accumulation of trehalose and glycogen [2], decreased transcription [3], [4] as well as translation [5] and specific cellular re-organization such as assembly of specific structures upon entry into quiescence [6], [7]. However, entry, maintenance and exit from the quiescent state are still poorly understood [1], [2], [8].

The ubiquitin-proteasome system is the major proteolytic mechanism in the cell and is crucial for cell proliferation [9]. It is also one pathway that is essential for survival in stationary phase [10], [11], [12]. The 26S proteasome is a multicatalytic protease that degrades polyubiquitinated proteins into short peptides [13]. In addition to its role as a protease, the proteasome also functions non-proteolytically in a variety of cellular processes, including transcription [14], [15], DNA repair [16] and chromatin remodeling [17]. The 26S proteasome is composed of two sub-complexes: a 20S core particle (CP) carrying the proteolytic activity and the 19S regulatory particle (RP). The 19S RP can be further dissociated into two sub-complexes referred to as the base and the lid [18]. The base made up of six homologous AAA-ATPases together with two non-ATPase subunits mediates a direct contact with the 20S core complex. The lid of the RP is made of nine non-ATPase subunits and contains a deubiquitinase activity carried by Rpn11. An additional subunit, Rpn10, connects the base to the lid. The main functions of the 19S RP are to recognize ubiquitinated proteins, to cleave the ubiquitin moiety and to unfold and insert the substrates into the 20S core particle [19].

In the yeasts *Saccharomyces cerevisiae* and *Schizosaccharomyces pombe*, it has been shown that the proteasome proteolytic activity decreases upon cell entry into stationary phase [10], [11], [12]. This effect did not correlate with a decrease of proteasome subunit abundance [6], [10] but rather with a disassembly of the 19S RP and 20S CP from the 26S holoenzyme as observed *in vitro* with crude extracts. The precise cellular role of the proteasome and the fate of disassembled subcomplexes in quiescent yeast cells remain obscure. However, it has been shown that the 26S proteasome, while localized diffusely into the nucleus of dividing cells, is reorganized *in vivo* when cells reached the stationary phase. Proteasome subunits have been shown to migrate first at the nuclear periphery and to rapidly co-localize into cytoplasmic structures named proteasome storage granules (PSGs [6]). This phenomenon is rapidly and completely reversed upon cell reentry into the proliferation cycle. Importantly, the proteasome subunits migration from cytosolic foci to the nucleus upon exit from quiescence occurs even in the absence of *de novo* protein synthesis [6]. The mechanisms leading to PSG formation and maintenance and whether they are required for cell longevity are completely unknown.

In order to elucidate how PSG are formed, we took advantage of a temperature-sensitive allele of the essential *RPN11* gene, named *rpn11-m1*, which specifically induces a proteasome assembly defect even at the permissive temperature [20]. Rpn11 is a deubiquitinating enzyme, and mutations in residues that contribute to its active catalytic site abolish function and cause lethality [21], [22]. The *rpn11-m1* mutation results in a truncated protein lacking the last 31 amino acids (replaced by nine illegitimate residues) that gives rise to proteasome proteolysis defect, aberrant mitochondrial morphology and proteasome [20], [21],[23],[24]. Strikingly, we uncovered a new functional domain of the carboxyl terminal part of Rpn11, which was recently found to consist of two long helices (49 and 29 residues) connected by a linker of six residues [25]. Overexpression of this domain *in trans* is able to overcome all the *rpn11-m1* defects [26]. We also used an intragenic revertant of *rpn11-m1*, previously named *rpn11-RevA5*, that carries seven amino acid changes in the carboxyl terminal domain and was previously shown to be able to restore growth at 36°C as wild-type cells but still harboring an abnormal mitochondrial network [24], [26].

Here we demonstrate by using these two different *rpn11* mutants that assembled-26S proteasomes are required for the formation of PSGs in stationary phase and that sub-complexes of proteasome cannot migrate into cytosolic foci in quiescent cells. Furthermore, we present evidence that the carboxyl terminal domain of the Rpn11 subunit plays a critical role in promoting 26S stability and in enabling cytosolic re-localization of proteasomes in quiescent cells.

## Results

### Proteasome Instability in *rpn11-m1* and *rpn11-m5* Cells

**Figure 1:**
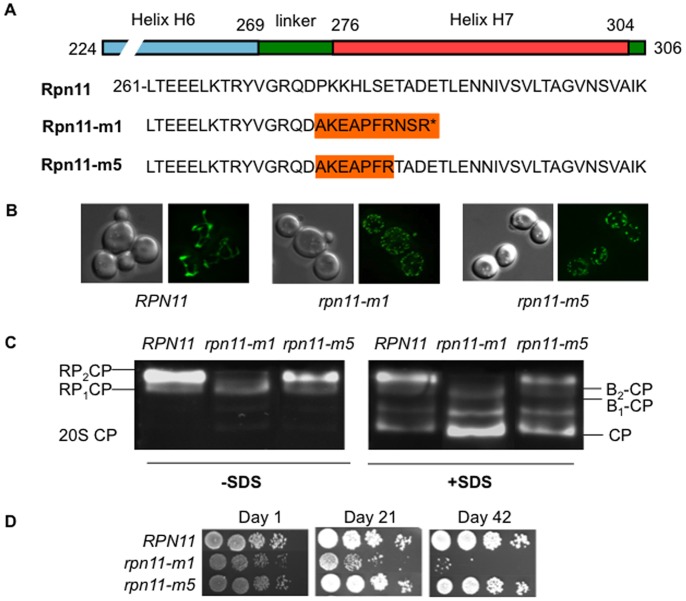
Phenotypes of the *rpn11-m1* and *rpn11-m5* mutant strains. (**A**) Alignment of the carboxyl amino acid sequences of Rpn11, Rpn11-m1 and Rpn11-m5. The amino acid changes are in orange. (**B**) Wild type (W303-1B background), *rpn11-m1* and *rpn11-m5* cells expressing mtGFP were grown to log phase in glucose containing medium (YPD) and examined by fluorescence (right) and phase contrast (left) microscopy. (**C**) Cell extracts prepared from exponentially growing wild type, *rpn11-m1* or *rpn11-m5* strains, brought to an identical cell density at the permissive temperature of 26°C were clarified by centrifugation, and samples containing identical amounts of total protein were separated by non-denaturing PAGE (native gels). Proteasomes were visualized by the fluorogenic peptide overlay assay. Proteasome holoenzymes in WT and *rpn11-m5* are found as a mixture of symmetric doubly capped (RP2CP) and asymmetric singly capped (RP1CP) conformations. 26S proteasomes in *rpn11-m1* are found almost exclusively in lidless forms (B2-CP or B1-CP). 20S CP is visualized upon activation of the CP by 0.05% SDS (right panel). Higher levels of dissociated free 20S CP are evident in *rpn11-m1* and more discrete for *rpn11-m5*. (**D**) Survival during starvation-induced stationary phase of wild type, *rpn11-m1* and *rpn11-m5* strains on glucose containing rich medium (YPD) at 26°C after growth for 1, 21 and 42 days. Comparable number of cells was spotted at 10-fold dilutions on YPD medium.

We first examined proteasome assembly of two *rpn11* mutants known as *rpn11-m1* and *rpn11-RevA5,* the latter renamed hereafter *rpn11-m5*. The *rpn11-m1* mutation is a frameshift in position 276 that results in a truncated protein lacking its C-terminal last 31 amino acids replaced by nine non-native residues (Figure 1A). The *rpn11-m5* allele is an intragenic suppressor of *rpn11-m1* able to rescue the cell cycle defect of *rpn11-m1* cells but not their mitochondrial morphology defect ([24], [26] and Figure 1B). This intragenic mutation restored the end of the open reading frame downstream Arginine 282 of *rpn11-m1* but maintained seven amino acid changes compared to the wild type sequence (Figure 1A). The *rpn11-m1* proteasome structural defect has been shown to be the source of *rpn11-m1* cell cycle phenotype [21] thus rescue of the cell cycle defect by *rpn11-m5* could result from a correct 26S proteasome assembly in this strain. Therefore, we monitored proteasome conformation by non-denaturating electrophoresis and in gel activity of crude extracts from wild type, *rpn11-m1* and *rpn11-m5* cells (Figure 1C). Proteasomes were visualized by the use of the fluorogenic peptide Succinyl-LLVY-AMC, a tetrapeptide substrate of the proteasome allowing the assessment of the proteolytic activity. The vast majority of proteasomes from exponentially growing wild type or *rpn11-m5* cells was found as doubly capped (RP_2_CP) or singly capped (RP_1_CP) 26S holoenzymes. In contrast, *rpn11-m1* proteasomes were almost exclusively present as lidless base–CP complexes as previously described ([20], [21], [23] and Figure 1C). Upon activation of the core particle by SDS, 20S CP can be visualized. Slightly higher levels of dissociated free 20S CP was observed in *rpn11-m5* compared to wild-type proteasomes indicating a lower stability of the *rpn11-m5* proteasomes *in vitro*. High level of free 20S CP was evident in *rpn11-m1* proteasomes as previously described [20].

In crude extracts, proteasome disassembly has been shown to correlate with loss of viability during stationary phase [10], [11], [12]. Therefore we examined the survival rate of wild type, *rpn11-m1* and *rpn11-m5* strains in stationary phase (Figure 1D). During the first week of starvation, the three strains maintained viability. However, after three weeks in stationary phase, an important drop in viability of *rpn11-m1* cells was observed whereas *rpn11-m5* and wild type cells remained viable even after 6 weeks in stationary phase.

Altogether, these results show that *rpn11-m5* proteasomes are stable enough to allow *rpn11-m5* cells to survive in stationary phase even with fragmented mitochondria whereas *rpn11-m1* cells containing mis-assembled proteasomes and fragmented mitochondria exhibit an important loss of viability in stationary phase.

### *rpn11-m1* Proteasome Subunits do not Migrate into Cytosolic Foci in Stationary Phase Cells

**Figure 2:**
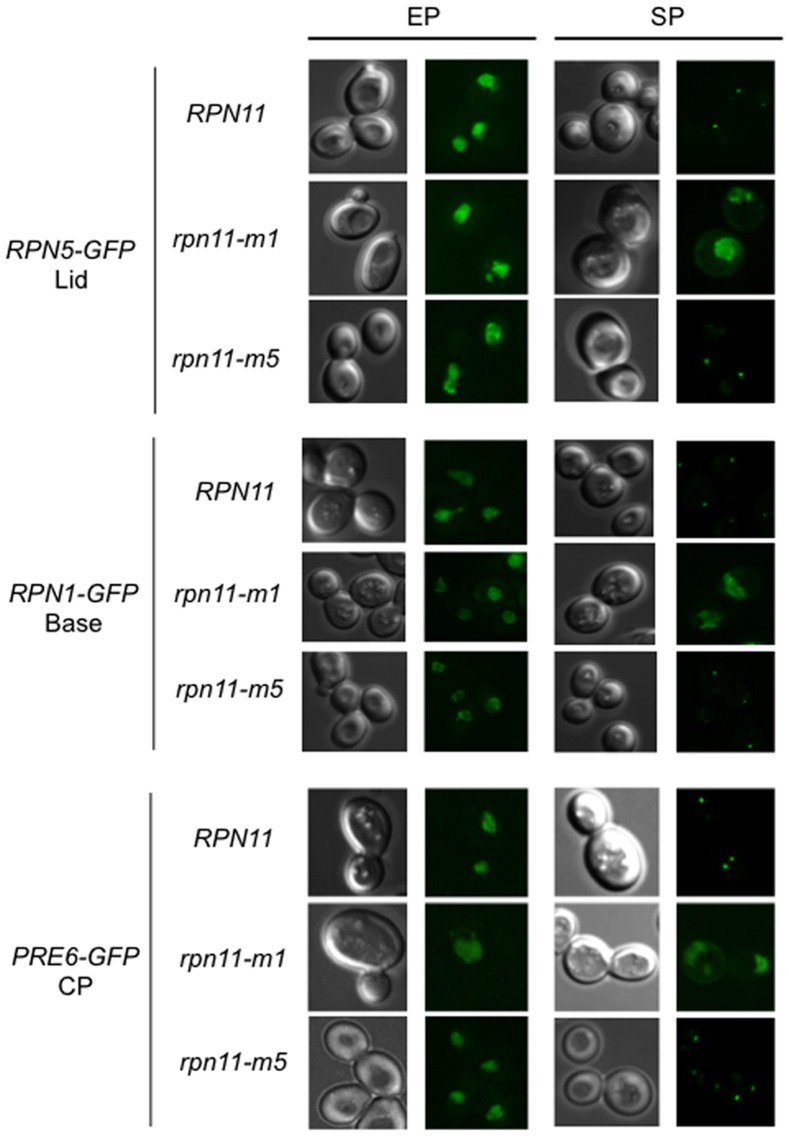
Proteasome subunits localization in exponential and stationary growth phases. Wild type, *rpn11-m1* and *rpn11-m5* cells expressing Rpn5-GFP (W303), Rpn1-GFP (W303) or Pre6-GFP (BY4741) were grown in glucose and adenine containing rich medium (YPDA) at 26°C and examined by fluorescence microscopy in the exponential growth phase (EP) and after 5 days in stationary phase (SP). Typical images of each subunit fused to GFP localization are shown. (CP/20S Core Particle).

It has been shown that upon entry into the stationary phase, all subunits of the 26S proteasome examined to date (nine for the 20S CP and eleven for the 19S RP) simultaneously migrated first to the nuclear periphery and then re-localized into PSGs [6]). We addressed whether PSGs could be formed in *rpn11-m1* and *rpn11-m5* cells when they reached the stationary phase. For this purpose, we followed the localization of three proteasome subunits: Rpn5 (19S RP, lid), Rpn1 (19S RP, base) and Pre6 (20S CP, α subunit) in living cells. For each subunit, GFP was fused at the C-terminus at the chromosome locus of the corresponding gene in the *rpn11-m1*, *rpn11-m5* and the isogenic wild-type strains. All strains expressing Rpn5-GFP, Rpn1-GFP or Pre6-GFP did not show any growth defect in exponential phase (data not shown). As previously shown [6], these three proteasome subunits were localized in the nucleus of wild-type proliferating cells and as cytosolic dots or PSGs in wild type quiescent cells (Figure 2). In actively dividing *rpn11-m1* and *rpn11-m5* cells, the three proteasome subunits examined were also localized in the nucleus. In stationary phase, the typical re-localization of these subunits into cytosolic dots was observed in the wild type and *rpn11-m5* cells (Figure 2). In contrast, no cytosolic dots were observed in *rpn11-m1* cells in stationary phase and Rpn5-GFP, Rpn1-GFP and Pre6-GFP signals stained the *rpn11-m1* nucleus and its periphery even after 5 days in stationary phase (Figure 2). From those experiments we conclude that, on the contrary of wild type and *rpn11-m5* cells, PSGs cannot be formed in *rpn11-m1* cells in stationary phase. Although 20S CP and lid-less proteasomes exist in *rpn11-m1* cells (Figure 1C), these sub-complexes were not able to form cytosolic dots in stationary phase.

### PSGs can be Formed in *Δrpn10*, *Δump1*, and *Δspg5* Proteasome Assembly Deficient Mutant Cells

**Figure 3:**
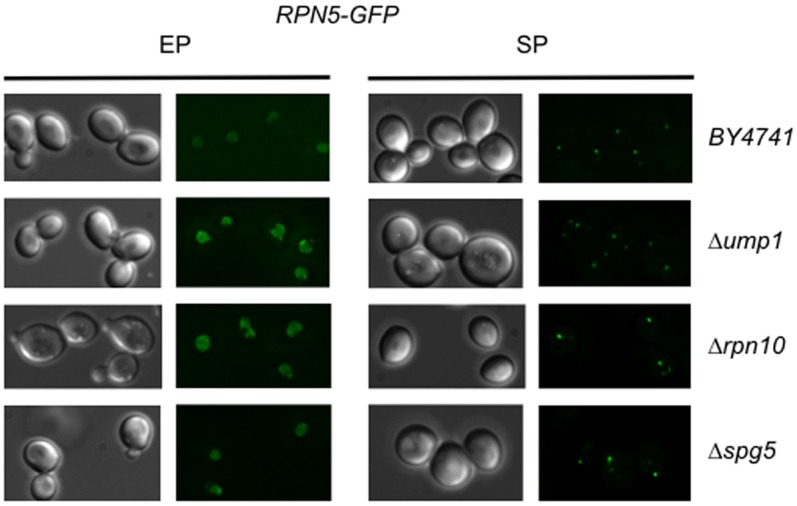
Rpn5-GFP localization in proteasome assembly defect mutant cells in exponential and stationary growth phases. Wild type, *Δump1*, *Δrpn10* and *Δspg5* cells expressing Rpn5-GFP were grown in glucose containing rich medium (YPD) at 26°C and examined by fluorescence microscopy in the exponential growth phase (EP) and after 5 days in stationary phase (SP). Typical images of Rpn5-GFP localization are shown.

We then assessed whether absence of PSG formation observed in *rpn11-m1* was specific to cells that display proteasome assembly defect. Therefore we examined PSG formation in the viable *Δrpn10* and *Δump1* deletion strains, known to exhibit proteasome assembly defect in dividing cells [18], [27], [28], [29], [30]. Rpn10 is a proteasome receptor of multiubiquitinated proteins that is also required for promoting proteasome stability. Apparent reduction in 26S holoenzyme level and higher level of dissociated free 20S CP were observed *in vitro* in *Δrpn10* crude extracts [18], [27]. The Ump1 protein is a proteasome maturation factor that is required for the proper coordination of proteasome assembly. A huge accumulation of free lid, indicative of base assembly defect, was found *in vitro* in *Δump1* crude extracts [29], [30]. We also examined PSG formation in *Δspg5* cells, for which proteasome structure defect has been evidence solely in quiescent cells [28]. Expression of Spg5 is induced in stationary phase where it binds transiently the 19S regulatory particle. *Δspg5* cells showed only 26S proteasomes with near complete absence of the other sub-complexes *in vitro* [28]. PSG formation in *Δrpn10, Δump1* and *Δspg5* cells has been followed by the subcellular localization of Rpn5-GFP in growing cells and after five days in stationary phase (Figure 3). Rpn5-GFP showed a nuclear localization in all the strains examined in dividing cells and a clear cytosolic localization as discrete foci characteristic of PSGs after five days spent in stationary phase. Altogether, these results indicate that defect in assembly of the 26S proteasome does not result necessarily in PSG formation impairment.

### Delayed Formation of PSGs in *rpn11-m5* Cells

**Figure 4:**
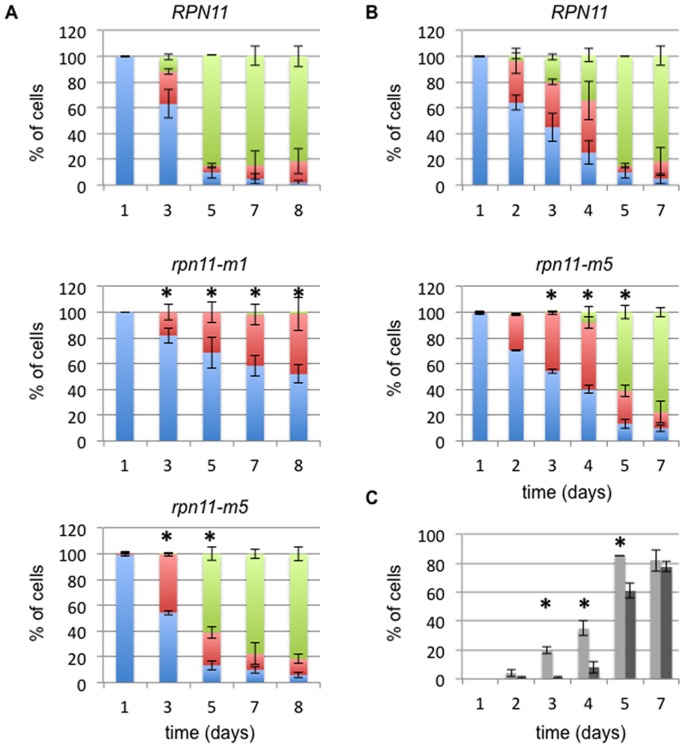
Localization of Rpn5-GFP in proteasome mutants defective in 26S assembly/stability. (**A**) Wild type, *rpn11-m1* and *rpn11-m5* cells expressing Rpn5-GFP were grown in YPDA medium at 26°C during 8 days. For each time point (day), the OD_600 nm_ was monitored, the survival rate performed (Figure S1) and the localization of Rpn5-GFP fluorescence was scored as nuclear (blue bar), at the nuclear periphery (red bar) or as cytosolic dots (green bar; n>100 cells for each time point; two independent experiments; error bars report the differences between the two experiments). (*) indicate that the differences in the distribution of the Rpn5-GFP signal in the mutant cells are significant relative to the wild-type cells after statistical analyses (Pearson’s chi-squared test, P values <0.05). (**B**) Wild type and *rpn11-m5* cells producing Rpn5-GFP were grown in YPDA medium at 26°C during 7 days. Localization of Rpn5-GFP was scored as in (A) but every day from day 1 to day 5 and at day 7. (**C**) Comparison of Rpn5-GFP-cytosolic foci apparition between the wild type (grey) and the *rpn11-m5* mutant (black) for each day. Error bars represent the difference observed between the two experiments and (*) indicates that the difference between the two strains is significant (Fisher’s exact test, P values <0.05).

We next followed the kinetics of Rpn5-GFP re-localization in the wild type, *rpn11-m1* and *rpn11-m5* strains grown in rich medium during eight days (Figure 4A). At the same time, OD_600nm_ was monitored and percentage of viable cells during the course of the experiment was evaluated (Figure S1). As previously observed, no cytosolic dots were formed in *rpn11-m1* cells even after 8 days of culture while PSGs were clearly formed after 5 days of culture in wild type and *rpn11-m5* cells (Figure 4A). However, in *rpn11-m5* cells, PSGs seemed to appear later in stationary phase if compared to wild type cells (Figure 4A). Same results were obtained when PSGs were followed with Rpn1-GFP instead of Rpn5-GFP (data not shown). By examining more systematically the time-dependent PSG formation in *rpn11-m5* cells (Figure 4B and 4C), a two days delay was required to observe cytosolic dots. Although most proteasomes are fully assembled *in vitro* (Figure 1C), the seven amino acid changes in Rpn11-m5 cause a delay in the formation of PSGs. An important drop in cell viability was observed for *rpn11-m1* cells unable to form PSGs whereas even if PSG formation is delayed in *rpn11-m5*, these cells remained as viable as wild type cells (Figure 1D).

### *rpn11-m1* Cells are Able to Enter into Quiescence

**Figure 5:**
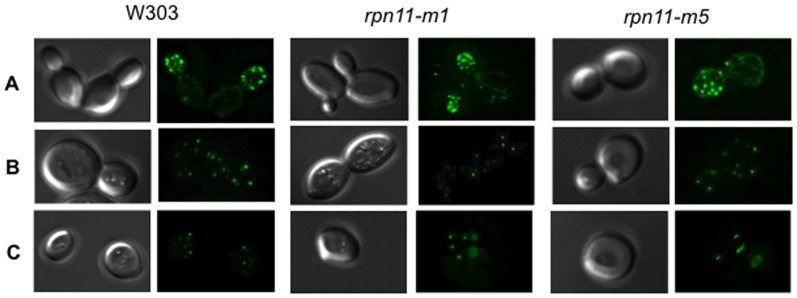
Actin cytoskeleton organization upon entry into the stationary phase. Wild type, *rpn11-m1* and *rpn11-m5* cells were grown in YPDA medium at 26°C. At various stage (**A**) exponential phase, (**B**) diauxic shift and (**C**) after five days in stationary phase, cells were taken off and stained with Alexa-phalloidin.

As PSGs are not formed in *rpn11-m1* cells in stationary phase, we asked whether this strain was able to entry into the quiescent state. For that purpose, we followed actin bodies formation, a specific actin-cytoskeleton organization observed specifically in starvation-induced stationary phase [7]. Proliferating cells grown in glucose rich medium display an actin cytoskeleton composed of three structures containing F-actin: actin cables, actin patches and an actin cytokinetic ring [31]. In stationary phase, actin patches disappear and a new actin organization called actin bodies appears [7]. Staining cells with phalloidin revealed that actin cables and actin patches were polarized to the active growth sites in wild type, *rpn11-m1* and *rpn11-m5* cells in exponential growth phase (Figure 5A). After glucose exhaustion (diauxic shift) actin cables became disorganized and actin patches remained depolarized in the three strains (Figure 5B). Finally, in stationary phase specific actin bodies could be observed in wild type, *rpn11-m1* and *rpn11-m5* cells (Figure 5C). This result indicates that *rpn11-m1* cells, unable to form PSGs, are able to form actin bodies in stationary phase, a hallmark of quiescent cells. Thus absence of PSG in *rpn11-m1* cells is not a consequence of a defective entry into quiescence.

### Overexpression *in trans* of the Rpn11-carboxyl Terminal Domain Restores PSGs Formation and Long-term Viability of *rpn11-m1* Cells

**Figure 6:**
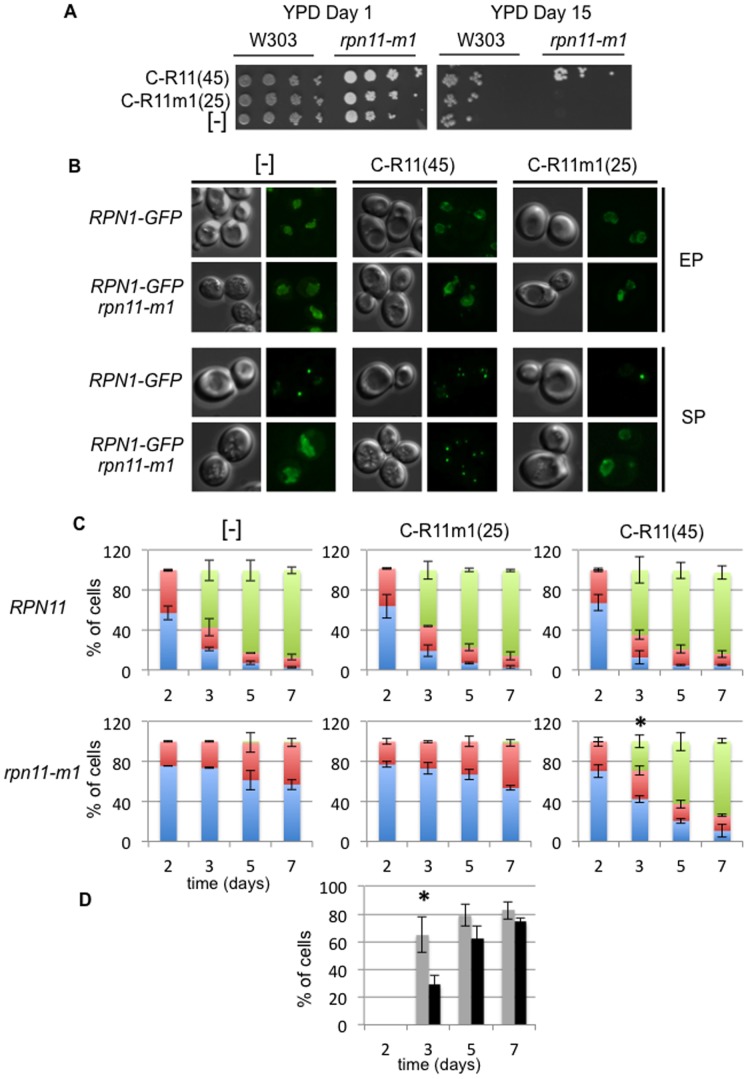
Overexpression of the Rpn11 carboxyl terminus. (**A**) Survival during starvation-induced stationary phase of wild type or *rpn11-m1* strains containing the plasmid overexpressing the 45 amino acids of the Rpn11 carboxyl terminus (C-R11(45)) or its mutated form (C-R11m1(25)) on rich medium (YPD) at 26°C after growth for 1 and 15 days in rich medium at 26°C. Comparable number of cells was spotted at 10-fold dilutions on YPD medium. (**B**) Wild type and *rpn11-m1* cells expressing Rpn1-GFP and overproducing C-R11(45) or C-R11m1(25) were grown in rich medium (YPDA) at 26°C and examined by fluorescence microscopy in the exponential growth phase (EP) and after 5 days in stationary phase (SP). Typical images of Rpn1-GFP localization are shown. (**C**) Wild type and *rpn11-m1* cells expressing Rpn1-GFP and overproducing C-R11(45) or C-R11m1(25) were grown in YPDA medium at 26°C. For each time point (day), the localization of Rpn1-GFP fluorescence was scored as nuclear (blue bar), at the nuclear periphery (red bar) or as cytosolic dots (green bar; n>100 cells for each time point; two independent experiments; error bars report the differences between the two experiments) and (*) indicate that the difference in the distribution of the Rpn1-GFP signal in the mutant cells are significant relative to the wild-type cells both overproducing C-R11(45) after statistical analyses (Pearson’s chi-squared test, P values <0.05). (**D**) Comparison of Rpn1-GFP-cytosolic foci apparition between the wild type (grey) and the *rpn11-m5* mutant (black) both overexpressing C-R11(45) for each day. Error bars represent the difference observed between the two experiments and (*) indicates that the difference between the two strains is significant (Fisher’s exact test, P values <0.05).

We previously showed that overexpression of the last carboxyl 100 amino acids of Rpn11, devoid of the Rpn11 catalytic domain, was able to rescue the cell cycle and mitochondrial morphology defects of *rpn11-m1* cells [24], [26]. In order to define more precisely the carboxyl domain required to fulfill the rescuing effect, we constructed and expressed different length of this domain (data not shown). We found that the last 45 amino acids, corresponding to the C-terminal segment of helix H6, which forms a coiled-coil with the helix H7 and comprises the linker (Figure 1), were necessary and sufficient to fully restore *in trans* a wild type phenotype (correction of temperature sensitivity and mitochondrial morphology) when overexpressed in *rpn11-m1* cells (Figure S2). We then examined the survival rate in stationary phase of wild type and *rpn11-m1* cells overproducing the wild type carboxyl-45aas domain or the equivalent carboxyl domain of *rpn11-m1* (C-R11(45) and C-R11m1(25) respectively, Figure 6A). Overexpression of these domains in wild-type cells did not induce any phenotype. However, overproducing C-R11(45) was able to restore the *rpn11-m1* growth defect at 36°C (Figure S2) and to rescue the *rpn11-m1* loss of viability in the stationary phase (Figure 6A). The overexpression of C-R11m1(25) had no effect on wild type and *rpn11-m1* cell viability in stationary phase. Importantly, survival of *rpn11-m1* cells expressing C-R11(45) in stationary phase was indistinguishable from the wild-type strain.

We next examined PSG formation in *rpn11-m1* cells overproducing these domains (Figure 6B and 6C). We followed the localization of Rpn1-GFP in the wild-type and *rpn11-m1* strains overexpressing C-R11(45) or the truncated and mutated form C-R11m1(25). Overproduction of these domains did not change the Rpn1-GFP localization in the wild-type strain either in exponential or stationary phases (Figure 6C). In contrast, the overproduction *in trans* of the wild type carboxyl-45 aas allowed PSG formation in *rpn11-m1* quiescent cells whereas overexpression of the mutated form did not (Figure 6B and 6C). These PSGs appeared rapidly after cells reached the stationary phase at day 3 however with their accumulation was slightly delayed in comparison to the wild type strain (Figure 6C and 6D). Taken together, these findings indicate that the Rpn11-carboxyl domain of 45 amino acids is required to enable PSG formation and cell survival of *rpn11-m1* quiescent cells.

### The Rpn11 Carboxyl-45 Amino Acids Domain Expressed Independently in *rpn11-m1* Cells Co-localizes with PSGs in Stationary Phase

**Figure 7:**
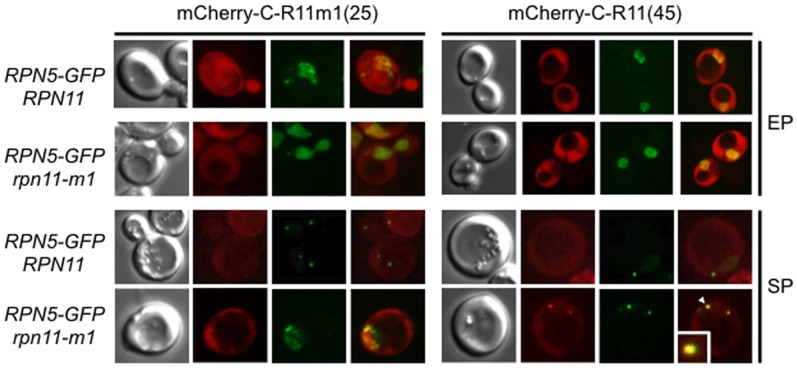
Co-localization of Rpn5-GFP and the mCherry fused to C-R11(45) or C-R11m1(25) in *rpn11-m1* cells. Wild type and *rpn11-m1* cells co-expressing Rpn5-GFP and the mCherry fused to the Rpn11 carboxyl terminus domain (mCherry-C-R11(45)) or to its mutated form (mCherry-CR11m1(25)) were grown in YPDA medium at 26°C and examined by fluorescence microscopy. Typical co-localization images obtained after one day of culture (exponential phase, EP) or after five days in stationary phase (SP). The white arrow in the last image points to a PSG, which is enlarged in the same image.

We then asked whether the 45 amino acids domain of Rpn11 would be found co-localizing with PSGs formed in *rpn11-m1* cells. For that purpose, we overexpressed the m-Cherry protein fused (N-terminal) to the 45 amino acids of the Rpn11-carboxyl terminus or its mutated form (m-Cherry-C-R11(45) and m-Cherry-CR11m1(25) respectively) in the wild-type or *rpn11-m1* cells expressing Rpn5-GFP (Figure 7). In dividing cells, Rpn5-GFP marked the nucleus and the m-Cherry-R11(45) and m-Cherry-R11m1(25) were present diffuse in the whole cell in both genetic contexts. When cells reached the stationary phase, Rpn5-GFP spots representing PSGs were formed in the wild type strain and no co-localization with the m-Cherry signal was observed. In *rpn11-m1* cells, overexpression of m-Cherry-CR11m1(25) did not allow PSG formation as expected. The Rpn5-GFP signal remained in or around the nucleus and the m-Cherry-CR11m1(25) signal was diffuse in the cytoplasm. In contrast, overexpression of m-Cherry-CR11(45) allowed PSG formation as revealed by Rpn5-GFP dots into the cytoplasm and the signal for m-Cherry-CR11(45), although still diffuse in the cytoplasm, was clearly concentrated in spots which co-localized with PSGs (Rpn5-GFP). These results indicate that the Rpn11-carboxyl domain of 45 amino acids is actively involved in PSG formation and strongly suggest its incorporation into the PSGs.

## Discussion

*In vitro* analyses from quiescent cell crude extracts have shown that 26S proteasomes are disassembled into their 20S core particle and 19S regulatory particle components [10]. However *in vivo*, most of the 19S and 20S subunits are simultaneously re-localized from the nucleus to discrete foci called PSGs when cells enter the stationary phase [6]. In the present study we bring evidence that in quiescent cells formation of PSGs require fully assembled 26S holoenzymes. We also found that proteasome subcomplexes such as 20S core particles and base-CP subcomplexes cannot be re-localized into cytosolic dots in quiescent cells. Furthermore, our findings point to a significant role of the Rpn11-carboxyl domain in the re-localization of 26S proteasomes into cytosolic PSGs in quiescent cells. Altogether, these results present evidence that the subcellular proteasome localization and proteasome function in quiescent cells play a critical role to maintain cell viability.

### PSG Formation

A particular mutation in *RPN11* (*rpn11-m1*) encoding the essential Rpn11 deubiquitinating enzyme, affects proteasome assembly [20], [21], [26] and interaction with multiubiquitinated proteins [23]. This well-characterized temperature sensitive *rpn11-m1* mutant contains a frameshift near the carboxyl terminus that results in the replacement of the terminal 31 amino acids by nine non-native residues. This mutant displays, as most of proteasome mutants, growth and proteolytic deficiencies at the non-permissive temperature. However at the permissive temperature, this mutant harbors specifically defects of the mitochondrial network [24], [26] and of proteasome assembly [20], [21], [23]. In permissive condition, the Rpn11-m1 protein is catalytically active but destabilizes proteasomes, *rpn11-m1* proteasomes being almost exclusively present as lidless base–CP complexes. The significant structural defect of *rpn11-m1* proteasomes led us to examine the behavior of *rpn11-m1* proteasomes *in vivo* at stationary phase. We found that *rpn11-m1* proteasomes, revealed by GFP fusion of subunits Rpn5, Rpn1 and Pre6 of the lid, the base and the core respectively, could not migrate to discrete cytosolic foci as PSGs in stationary phase even though the *rpn11-m1* cells were able to form actin bodies, a hallmark of quiescent cells. Thus absence of PSG in *rpn11-m1* cells is not a consequence of a defective entry into quiescence. Interestingly, although 20S CP and lid-less proteasomes exist, these proteasome sub-complexes were not able to form cytosolic foci in stationary phase. Therefore, it seems unlikely that PSGs contain proteasome subunits stuck together. Because the *rpn11-m1* cells display an important defect in proteasome assembly, we addressed whether other mutant cells also exhibiting proteasome structure anomalies could form PSGs in stationary phase. As *Δrpn10, Δump1* and *Δspg5* cells are able to form PSGs (Figure 3), it suggests that defect in 26S assembly/stability is not sufficient to abolish PSG formation and strengthens a significant role of Rpn11 in this process.

Another *rpn11* mutant, *rpn11-m5* which led to seven amino acid changes in the carboxyl domain of Rpn11, isolated as an intragenic suppressor of *rpn11-m1* able to rescue the *rpn11-m1* cell cycle defect but not the mitochondrial morphology defect ([24], [26] and Figure 1B) displays 26S proteasome amounts near to the wild type level. Although most 26S proteasomes are assembled, the seven amino acid changes in Rpn11-m5 cause a delay in PSG formation. However, a higher level of dissociated free 20S CP was observed in *rpn11-m5* compared to wild-type proteasomes (Figure 1C) suggesting that the kinetics of PSG formation could be correlated with the slight 26S instability observed *in vitro* in *rpn11-m5* crude extracts.

Because the Rpn11-m1 protein is catalytically active but destabilizes proteasomes, it has been proposed that the carboxyl terminal domain of Rpn11 promotes proteasome stability [20], [23], [25]. Here, we found that the carboxyl-45 amino acids of Rpn11 can function *in trans* to fully ensure growth at high temperature, to restore a wild type mitochondrial network (Figure S2) and to allow PSG formation and survival of quiescent *rpn11-m1* cells (Figure 6). PSGs formation in *rpn11-m1* overproducing the 45 carboxyl domain seems to be moderately delayed if compared to the wild-type strain, however PSG formation has been examined when cells reached the stationary phase in rich medium and we cannot ruled out that the plasmid encoding the carboxyl domain *in trans* was lost in some cells. Interestingly, this domain was found *in vivo* to co-localize with PSGs (Figure 7). This result is consistent with the fact that the carboxyl terminus of Rpn11 is accessible from the outside of the proteasome as shown by a cryo-EM structure of the 26S proteasome [25], [32]. These findings strongly suggest that PSGs can only be formed in *rpn11-m1* when 26S proteasomes contain the Rpn11 carboxyl domain added *in trans*, which leads to stable and functional 26S proteasomes.

Fully assembled-26S proteasomes seem to be required for PSG formation in quiescent cells however several *in vitro* studies showed a reduction of proteolysis activity and a disassembly of the 26S proteasomes in quiescent cell crude extracts [10], [11], [12]. Together, these data suggest that after migration of 26S proteasomes into cytosolic foci, 26S proteasomes dis-assembled and may be maintained as such. One factor, Spg5 whose expression is induced in stationary phase, may play a role in this process as it has been recently shown *in vitro* that *Δspg5* quiescent cells contain only 26S proteasomes with no detectable 19S and 20S subcomplexes [28].

### Long-term Survival in Stationary Phase and PSG Formation

The *rpn11-m1* cells present four main phenotypes at the permissive temperature: *in vitro* mis-assembled 26S proteasome [20], [23], short lifespan in stationary phase (this study), incapacity to form PSGs (this study) and fragmented mitochondria [20], [24], [26], [33], [34]. The decrease in survival of *rpn11-m1* cells in quiescent state does not seem to be a consequence of PSG absence since *Δump1* [11], [35], *Δspg5* [28] and catalytic proteasome mutant cells, all exhibiting short lifespan in stationary phase [10], [11], [12], are able to form PSGs (Figure 3). As mitochondrial functions have been shown to be critical for survival in stationary-phase [36], the mitochondrial defects of *rpn11-m1* cells (fragmented mitochondria) could be also responsible for *rpn11-m1* short chronological lifespan. However, the *rpn11-m5* mutant cells that still harbors fragmented mitochondria do survive in stationary phase as well as the wild type cells. Altogether, these results add evidence that the 26S proteasome function in quiescent cells play a primary and critical role to sustain viability in stationary phase according to previous data [10], [11], [12] and that subcellular 26S proteasome localization in stationary phase may rather be critical for the rapid exit from the quiescent state when nutrients become available.

What drives 26S proteasomes to their final destination in stationary phase (from nucleus to cytosolic PSGs) and how it occurs remain important opened questions. Here we present evidence that the Rpn11 protein and more probably its carboxyl domain may play a critical role in the formation of PSGs. Indeed, none of the subunits from the lid (Rpn5), the base (Rpn1) and the core 20S (Pre6) examined in *rpn11-m1* cells, were able to reach the cytosol but rather seemed to be stuck in and around the nucleus even if proteasomes are in lid-less conformation. This raises interesting questions. Do proteasomes exit the nucleus only under 26S conformation? How this huge complex passes through the nuclear envelop? Interestingly, during the formation of PSGs, proteasomes have been detected as intense immobile dots close to the nuclear periphery before being dots moving into the cytoplasm [6]. The *rpn11-m1* proteasomes never form dots even close to the nuclear periphery suggesting that this step is impaired in *rpn11-m1* cells.

### Rpn11 a Key Player in Proteasome Destiny

Numerous studies have suggested that proteasome impairment promotes a variety of age-related events and could promote age-related cytotoxicity. A decline of proteasome activity correlated to aging has been observed beyond *S. cerevisiae* and *S. pombe*, in human, other mammals [37], and aged flies [38]. In *Drosophila melanogaster*, reduction of the 26S proteasome activity was shown to be associated with impaired assembly of the 26S proteasome during aging [39]. Interestingly, overexpression of *D. melanogaster* Rpn11 extends the life span by preventing reduction of the 26S proteasome activity. Conversely, the loss of function of Rpn11 enhances age-related reduction of the 26S activity and leads to a shorter life span of the fly [39]. Thus, together with our results, the Rpn11 subunit seems to play a significant and critical role as a potential regulator of 26S assembly, function and sub-cellular localization that goes far beyond its role as a deubiquitinase.

## Materials and Methods

### Strains and Plasmids

Strains used in this study were derived from the W303 or BY4741 genetic contexts and the same results were obtained. Strains are listed in table 1. Standard techniques were used for strain constructions [40] and transformations. The RPN5-GFP::TRP1-MX6 or RPN5-GFP::HIS3-MX6 and RPN1-GFP::HIS3-MX6 cassettes have been used to introduce GFP at the chromosomal RPN5 and RPN1 loci in either W303 or BY4741 genetic contexts. Yeasts were cultured at 26°C in YPD medium consisting of 1% yeast extract, 2% bacto peptone and 2% glucose. Viability of the cells in culture was assessed by counting the colony-forming unit (CFU) from an average of 100 cells plated on 3 Petri dishes. The optical density at 600 nm (OD_600nm_) was analyzed at the indicated time. Survival in stationary phase was tested by inoculating cells from indicated strains in glucose containing rich medium (YPD or YPD plus adenine) at one OD_600nm_ (Day1). At the indicated days one OD_600nm_ of cells was serial diluted and spotted on Petri dishes containing rich medium.

**Table 1:** Strains used in this study.

Strain name	Genotype	Source
*RPN11 (W303-1B)*	*MATα leu2-3,112 trp1-1 can1-100 ura3-1 ade2-1 his3-11,15 RPN11∶3HA-KANMX6*	[24]
*rpn11-m1 (W303-1B)*	*MATα leu2-3,112 trp1-1 can1-100 ura3-1 ade2-1 his3-11,15 rpn11-m1∶3HA-KANMX6*	[24]
*rpn11-m5 (W303-1B)*	*MATα leu2-3,112 trp1-1 can1-100 ura3-1 ade2-1 his3-11,15 rpn11-m5∶3HA-KANMX6*	This study
*RPN11 RPN5-GFP (W303-1B)*	*MATα leu2-3,112 trp1-1 can1-100 ura3-1 ade2-1 his3-11,15 RPN11∶3HA-KANMX6, RPN5:GFP(S65T)-TRP1*	This study
*rpn11-m1 RPN5-GFP (W303-1B)*	*MATα leu2-3,112 trp1-1 can1-100 ura3-1 ade2-1 his3-11,15 rpn11-m1∶3HA-KANMX6, RPN5:GFP(S65T)-TRP1*	This study
*rpn11-m5 RPN5-GFP (W303-1B)*	*MATα leu2-3,112 trp1-1 can1-100 ura3-1 ade2-1 his3-11,15 rpn11-m5∶3HA-KANMX6, RPN5:GFP(S65T)-TRP1*	This study
*RPN11 RPN1-GFP (W303-1B)*	*MATα leu2-3,112 trp1-1 can1-100 ura3-1 ade2-1 his3-11,15 RPN11∶3HA-KANMX6, RPN1:GFP(S65T)-HIS3*	This study
*rpn11-m1 RPN1-GFP (W303-1B)*	*MATα leu2-3,112 trp1-1 can1-100 ura3-1 ade2-1 his3-11,15 rpn11-m1∶3HA-KANMX6, RPN1:GFP(S65T)-HIS3*	This study
*rpn11-m5 RPN1-GFP (W303-1B)*	*MATα leu2-3,112 trp1-1 can1-100 ura3-1 ade2-1 his3-11,15 rpn11-m5∶3HA-KANMX6, RPN1:GFP(S65T)-HIS3*	This study
*RPN11 (BY4741)*	*MATa his3Δ1 leu2Δ0 met15Δ0 ura3Δ0 RPN11∶3HA-KANMX6*	This study
*rpn11-m1 (BY4741)*	*MATa his3Δ1 leu2Δ0 met15Δ0 ura3Δ0 rpn11-m1∶3HA-KANMX6*	This study
*rpn11-m5 (BY4741)*	*MATa his3Δ1 leu2Δ0 met15Δ0 ura3Δ0 rpn11-m5∶3HA-KANMX6*	This study
*PRE6-GFP (BY4741)*	*MATa his3Δ1 leu2Δ0 met15Δ0 ura3Δ0 PRE6:GFP-HIS3MX6*	Invitrogen
*RPN11 PRE6-GFP*	*MATa his3Δ1 leu2Δ0 lys2Δ0 ura3Δ0 PRE6:GFP-HIS3MX6, RPN11∶3HA-KANMX6*	This study
*rpn11-m1 PRE6-GFP*	*MATa his3Δ1 leu2Δ0 ura3Δ0 PRE6:GFP-HIS3MX6, rpn11-m1∶3HA-KANMX6*	This study
*rpn11-m5 PRE6-GFP*	*MATa his3Δ1 leu2Δ0 ura3Δ0 PRE6:GFP-HIS3MX6, rpn11-m5∶3HA-KANMX6*	This study
*RPN5-GFP (BY4741)*	*MATa his3Δ1 leu2Δ0 met15Δ0 ura3Δ0 RPN5:GFP-HIS3MX6*	This study
*RPN5-GFP Δump1 (BY4741)*	*MATa his3Δ1 leu2Δ0 met15Δ0 ura3Δ0 Δump1::KAN, RPN5:GFP(S65T)-HIS3*	This study
*RPN5-GFP Δrpn10 (BY4741)*	*MATa his3Δ1 leu2Δ0 met15Δ0 ura3Δ0 Δrpn10::KAN, RPN5:GFP(S65T)-HIS3*	This study
*RPN5-GFP Δspg5 (BY4741)*	*MATa his3Δ1 leu2Δ0 met15Δ0 ura3Δ0 Δspg5::KAN, RPN5:GFP(S65T)-HIS3*	This study
*Δump1 (BY4741)*	*MATa his3Δ1 leu2Δ0 met15Δ0 ura3Δ0 ump1::KAN*	Invitrogen
*Δrpn10 (BY4741)*	*MATa his3Δ1 leu2Δ0 met15Δ0 ura3Δ0 rpn10::KAN*	Invitrogen
*Δspg5 (BY4741)*	*MATa his3Δ1 leu2Δ0 met15Δ0 ura3Δ0 spg5::KAN*	Invitrogen

Plasmids expressing either C-R11(45), C-R11-m1(25) with or without the mCherry in amino-terminal position were generated by PCR and cloned under the *PGK1* promoter into the BFG1 plasmid (2 µ, *LEU2*). All the amplified DNAs were verified by sequencing.

### Native Crude Extract and Native Gel Analysis

Cells were grown in YPD until the OD_600nm_ has reached 0.8–1. Cells were harvested and resuspended in prechilled (4°C) buffer E (50 mM Tris pH 7.5, 150 mM NaCl, 10% glycerol (v/v), 2 mM ATP, 1 mM DTT, 10 mM MgCl_2_). The frozen pellet was passed through the French cell press and the crude extract was spun 10 minutes at 13 000 g in a prechilled centrifuge. Supernatant was collected and the quantity of proteins was estimated by the Bradford method. Proteins were run on a native polyacrylamide gel as followed. Samples containing identical amounts of total protein were separated by non-denaturing PAGE 3.8% polyacrylamide gel (0,18 M Tris-borate pH 8.3, 5 mM MgCl_2_, 1 mM ATP, 1 mM DTT, 3,8% acrylamide). Gels were run for 2 hours at 4°C in the running buffer (0,18 M Tris-borate pH 8.3, 5 mM MgCl_2_, 1 mM ATP, 1 mM DTT). For in gel peptidase activity assay with Suc-LLVY-AMC, the native gels were incubated 20–25 minutes in a dark chamber in the presence of buffer G (50 mM Tris pH 7.5, 150 mM NaCl, 10% glycerol (v/v), 1 mM ATP, 1 mM DTT, 0,1 mM Suc-LLVY-AMC) and photographed. Native gels were further incubated in the presence of 0,05% SDS for 20 minutes to induce complete opening of the 20S gate.

### Fluorescence Microscopy, Cell Classification and Image Treatment

For live imaging, yeast cells were grown at 26°C in YPD supplemented with Adenine (YPDA) for the W303 genetic background. Aliquot of cells were washed and resuspended in PBS, immediately transferred to slides and imaged at 26°C. For visualization of mitochondria, cells were transformed with the pYX142-mtGFP plasmid [41], which expresses GFP fused to a mitochondrial import sequence.

Actin network was visualized by phalloidin staining of cells taken in the exponential phase, at the diauxic shift or in stationary phase. Cells were fixed in 3.7% formaldehyde directly added to the medium for 30 minutes at room temperature and then washed twice with PBS and resuspended in PBS containing 1% BSA. For cells in stationary phase an additional wash was performed in PBS with 1% triton and incubated for 15 minutes. Cells were further incubated 20 minutes in the presence of 2% triton and 0.033 µM green-phalloidin 488 (Molecular Probes), washed in PBS, and spotted onto slides.

For cell classification fluorescence was analyzed in single cells. When the proteasome signal was detected diffusely within the nucleus, cells were scored as “nuclear”. When the proteasome signal was clearly absent within the nucleus but at or around the nuclear periphery, cells were scored as “nuclear periphery”. Finally, as soon as the proteasome signal appeared as an intense dot, cells were scored as “PSG”. In each quantification experiment, at least 200 cells were counted (100 cells from two independent experiments).

Slides were examined with a DMIRE2 microscope (Leica, Deerfield, IL). Images were captured using a CCD camera (Roper Scientific, Tucson, AZ). Metamorph software (Universal Imaging, West Chester, PA) was used to deconvolute Z-series and treat the images.

## Supporting Information

Figure S1 Growth curves and cell survival measurements of wild type, *rpn11-m1* and *rpn11-m5* cells expressing Rpn5-GFP. Cells were grown in YPDA medium at 26°C during 8 days and used to monitor Rpn5-GFP localization of Figure 4. Growth curves of two independent experiments are shown in blue and red.
(TIF)

Figure S2 Complementation assays by the Rpn11 C-terminal domain added in *trans*. **(A)** Growth of the wild type or *rpn11-m1* strains transformed with the plasmid overproducing either the Rpn11 carboxyl domain of 45 amino acids (C-R11(45)), its mutated form (C-R11-m1(25)) or the empty plasmid (−). Cells were grown in liquid minimum medium and comparable number of cells were spotted at 10-fold dilutions on YPD medium and incubated at 26°C and 36°C. **(B)** Wild type and *rpn11-m1* cells expressing mtGFP and overproducing either C-R11(45), C-R11m1(25) or nothing (−) were grown to log phase in rich medium and examined by phase contrast (left) and fluorescence (right) microscopy.
(TIF)
