# Supplementary figures and images for: Integrity of the Saccharomyces cerevisiae Rpn11 Protein Is Critical for Formation of Proteasome Storage Granules (PSG) and Survival in Stationary Phase
Source: PLoS One. 2013 Aug 6;8(8):e70357. doi: 10.1371/journal.pone.0070357 (PMC3735599; doi:10.1371/journal.pone.0070357)

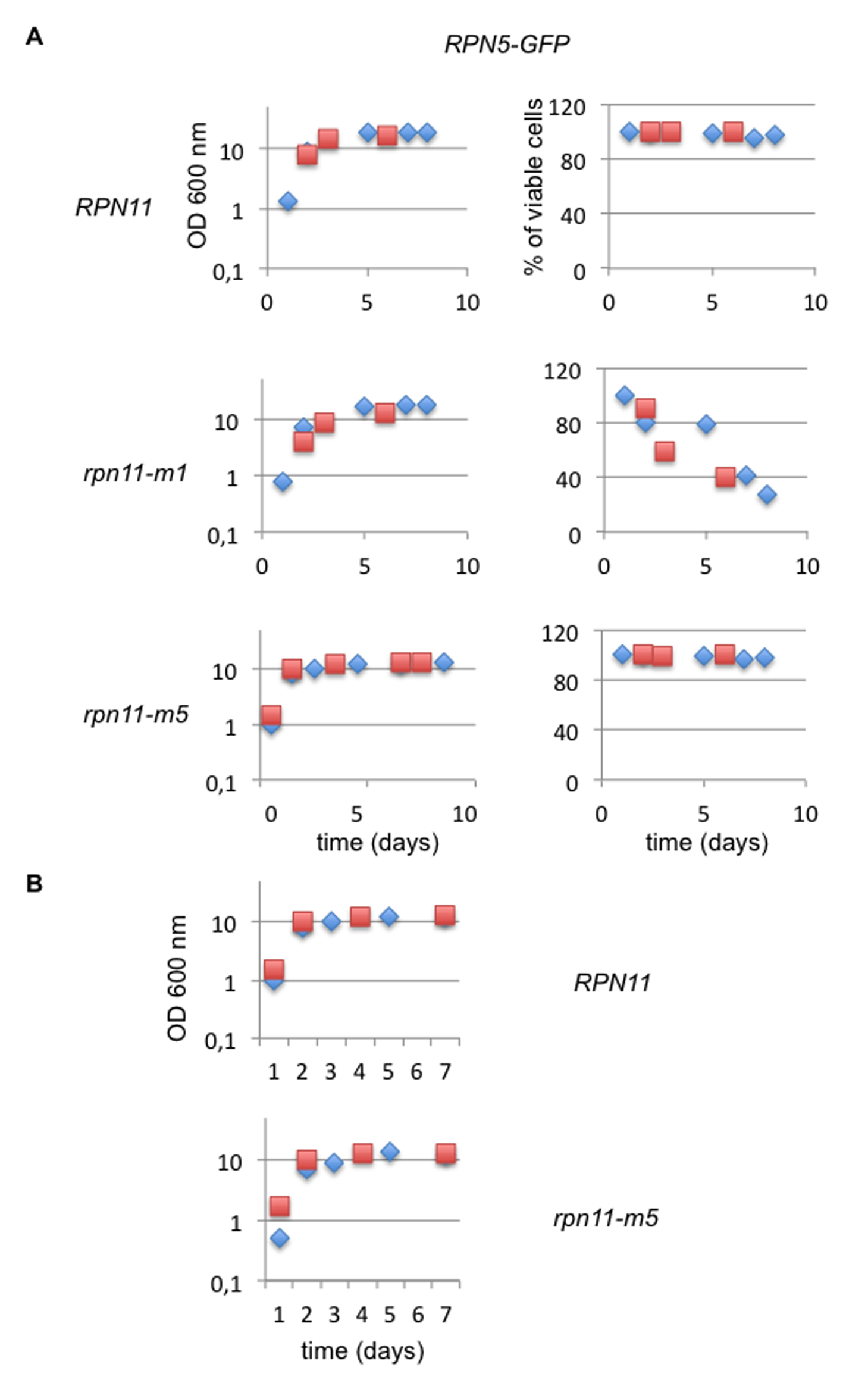

Supplement: Figure S1 — Growth curves and cell survival measurements of wild type, rpn11-m1 and rpn11-m5 cells expressing Rpn5-GFP. Cells were grown in YPDA medium at 26°C during 8 days and used to monitor Rpn5-GFP localization of Figure 4. Growth curves of two independent experiments are shown in blue and red. (TIF) [file pone.0070357.s001.tif]

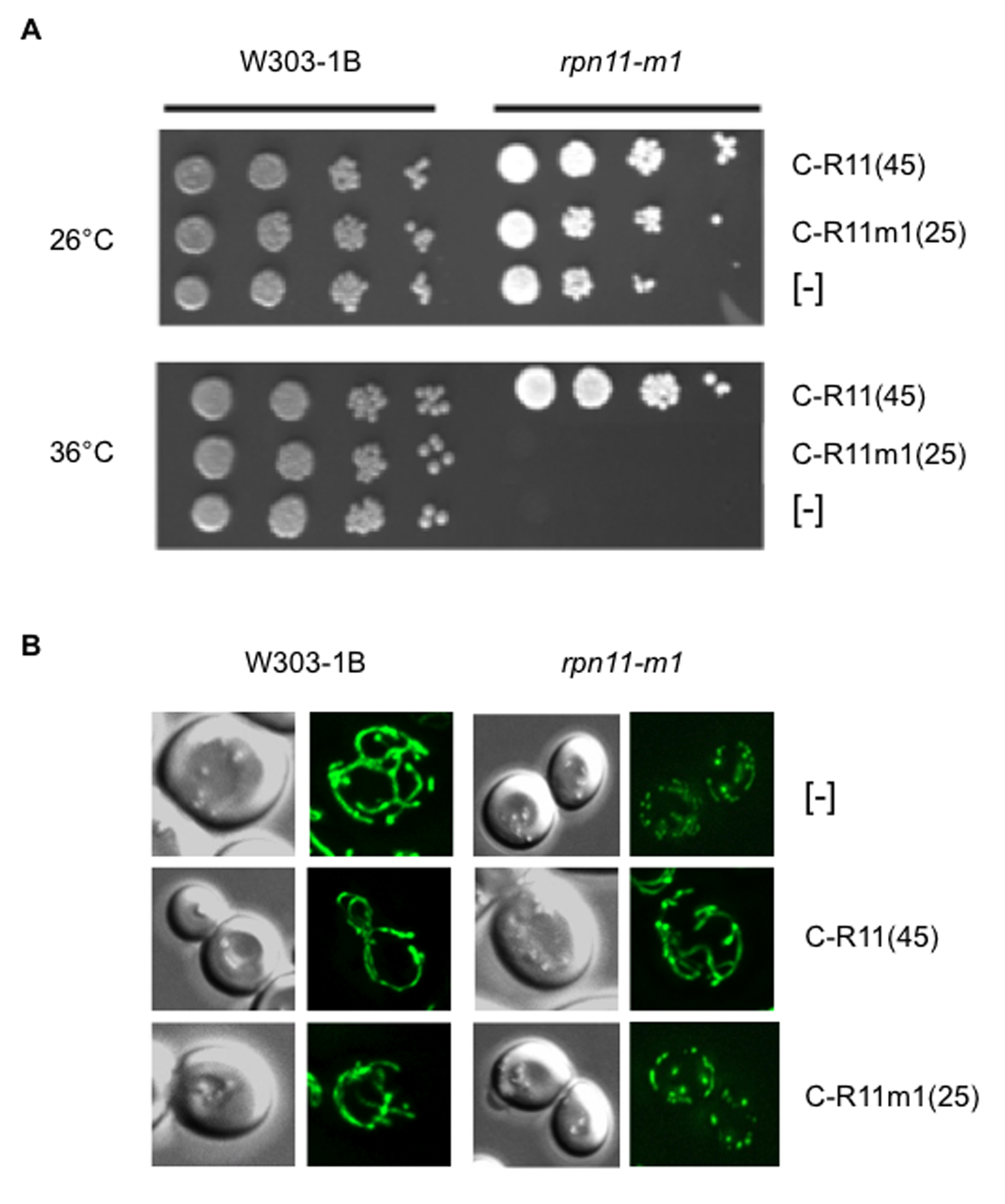

Supplement: Figure S2 — Complementation assays by the Rpn11 C-terminal domain added in trans. (A) Growth of the wild type or rpn11-m1 strains transformed with the plasmid overproducing either the Rpn11 carboxyl domain of 45 amino acids (C-R11(45)), its mutated form (C-R11-m1(25)) or the empty plasmid (−). Cells were grown in liquid minimum medium and comparable number of cells were spotted at 10-fold dilutions on YPD medium and incubated at 26°C and 36°C. (B) Wild type and rpn11-m1 cells expressing mtGFP and overproducing either C-R11(45), C-R11m1(25) or nothing (−) were grown to log phase in rich medium and examined by phase contrast (left) and fluorescence (right) microscopy. (TIF) [file pone.0070357.s002.tif]
